# The gut and circulating virome: emerging players in aging and longevity

**DOI:** 10.3389/fragi.2025.1731621

**Published:** 2026-02-09

**Authors:** Gretta Veronica Badillo-Pazmay, Carlo Fortunato, Laura Cianfruglia, Federica Novazzi, Pietro Giorgio Spezia, Luigi Rosa, Dolores Limongi, Carla Prezioso, Valeria D’Argenio, Olga Scudiero, Lisa Bevilacqua, Marco Malavolta, Patrizia Russo, Fabrizio Maggi, Marta Balietti, Robertina Giacconi

**Affiliations:** 1 Advanced Technology Center for Aging Research, IRCCS INRCA, Ancona, Italy; 2 Department of Medicine and Technological Innovation, University of Insubria, Varese, Italy; 3 Laboratory of Microbiology, ASST Sette Laghi, Varese, Italy; 4 Laboratory of Virology, National Institute for Infectious Diseases, Lazzaro Spallanzani-IRCCS, Rome, Italy; 5 San Raffaele Open University, Department of Human Sciences and Promotion of the Quality of Life, Rome, Italy; 6 Laboratory of Microbiology, IRCCS San Raffaele Roma, Rome, Italy; 7 CEINGE-Biotecnologie Avanzate “Franco Salvatore”, Napoli, Italy; 8 Department of Molecular Medicine and Medical Biotechnologies, University of Naples Federico II, Napoli, Italy; 9 Task Force on Microbiome Studies, University of Naples Federico II, Naples, Italy; 10 Center for Neurobiology of Aging, IRCCS INRCA, Ancona, Italy; 11 Department of Clinical and Molecular Sciences, Università Politecnica delle Marche, Ancona, Italy; 12 Clinical and Molecular Epidemiology, IRCCS San Raffaele Roma, Rome, Italy

**Keywords:** aging, centenarians, inflammaging, longevity, viroma

## Abstract

A growing body of evidence indicates that the human virome, comprising both the gut and circulating viral communities, plays a critical role in shaping host physiology across the lifespan. In the context of aging, this complex viral ecosystem is increasingly recognized as a key modulator of immune function, inflammation, and metabolic balance, with direct implications for healthspan and longevity. While much attention has traditionally focused on bacterial components of the microbiota, recent advances in metagenomics have uncovered age-related shifts in the composition and function of the virome, including expansion of specific bacteriophage families, reactivation of latent viruses, and the persistence of commensal viral pathobionts. These changes are tightly linked to immunosenescence, chronic inflammation, and neurodegeneration, hallmarks of unhealthy aging. Notably, centenarians appear to harbor a unique virome signature marked by increased viral diversity, enhanced lytic activity, and the enrichment of phage-encoded metabolic functions, suggesting a potential protective role in extreme longevity. Despite these insights, significant challenges remain in virome profiling, including technical biases, database limitations, and the vast proportion of taxonomically unassigned sequences known as “viral dark matter”. This review highlights emerging data on the aging virome, underscores its relevance within the Geroscience framework, and discusses current barriers and future directions for translating virome research into clinical aging studies.

## 1 Introduction

In mammals, the gut microbiota represents a vast and complex ecosystem of microorganisms, including bacteria, fungi, viruses, and archaea, that inhabit the digestive tract. These microbes play essential roles in maintaining intestinal homeostasis, modulating host immunity, and regulating metabolic processes (Jazayeri et al., 2017; Chioma et al., 2021). Among them, the bacteriome constitutes the most functionally characterized component, revealing important differences between healthy and diseased individuals, as well as between young and older adults (Tan et al., 2021; Mayneris-Perxachs et al., 2022; Ritz et al., 2024; Francini et al., 2025; Govender and Ghai, 2025).

However, in recent years, scientific interest in the gut virome, the collection of all viruses and viral genomes present in the gut, has grown considerably. This viral community includes bacteriophages that infect bacteria, eukaryotic viruses, and free viral nucleic acids (Guerin et al., 2018; Shkoporov, 2019). As an integral part of the gut microbiota, the virome contributes to intestinal and immune homeostasis, and the modulation of local immune responses along with the limitation of bacterial pathobiont expansion may also support gut barrier integrity (Buret, 2023). Nonetheless, not all components of the virome are beneficial. While the role of bacteriophages in pathobiont-mediated diseases is increasingly recognized (Fujimoto and Daichi, 2022), the concept of viral pathobionts, viruses that normally coexist with the host but may become immunopathogenic under certain conditions, remains less well defined and is only beginning to be explored. In this context, Riley (Riley, 2004) proposed that phages released into the intestinal lumen or other mucosal surfaces, particularly during dysbiosis or following environmental triggers, may aberrantly activate the immune system and contribute to autoimmune or inflammatory disorders. This hypothesis aligns with the broader view that the composition and activity of the gut virome can profoundly influence host physiology and immune regulation (Mills et al., 2013). Accordingly, a growing body of evidence supports a role for virome alterations in the onset and progression of various inflammatory diseases (Gao et al., 2021; Yang et al., 2021; Fujimoto and Daichi, 2022), with specific bacteriophages potentially exerting proinflammatory effects (Varadan and Grasis, 2025).

Recognizing its impact on host physiology, the virome is now being investigated within the framework of the Geroscience hypothesis, which seeks to elucidate the biological mechanisms underlying aging and age-related diseases (Moffitt, 2020). Recent findings by Francini and colleagues (2025) suggest that the gut microbiota of older adults is characterized by a decline in *Firmicutes* and an increase in *Bacteroidetes* and *Proteobacteria*. This shift results in a lower *Firmicutes*-to-*Bacteroidetes* ratio, often linked to gut dysbiosis. Notably, this altered ratio has been associated with a higher abundance of *Microviridae*, a family of bacteriophages, suggesting a potential virome signature of microbial imbalance. In the context of disease, Yang and colleagues (2021) demonstrated that obesity is associated with significant alterations in viral taxonomic composition and weakened viral-bacterial correlations, with these changes being further exacerbated in individuals with Type 2 Diabetes Mellitus. Furthermore, Lai et al. (2023) reported that shifts in gut microbial and viral composition, along with alterations in plasma metabolites and inflammatory mediators, may contribute to pre-hepatic disorders and foster a microenvironment conducive to hepatocellular carcinoma development in older adults. Notably, dysbiosis of the gut virome has also been linked to cognitive deficits (Mayneris-Perxachs et al., 2022).

**FIGURE 1:**
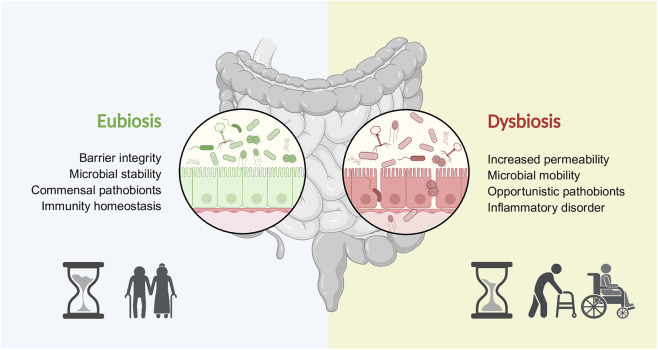
The impact of variation in virome stability in older adults. During aging, the maintenance of a healthy gut virome (eubiosis) supports key processes that not only extend lifespan but also promote a longer healthspan. Conversely, the onset of dysbiosis disrupts these protective functions, triggering harmful phenomena that reduce longevity and increase the time spent living with age-related diseases and reduced autonomy.

Since gut virome composition and function influence key processes of healthy aging (Figure 1), it is increasingly recognized as both a biomarker and a promising therapeutic target for promoting longevity and extending healthspan. This review summarizes current knowledge on the gut virome in relation to homeostasis and aging, with a focus on its interactions with the bacteriome, the host immune system, and the brain. We also explore potential contributions of extraintestinal viral communities, especially components of the circulating virome, and discuss key challenges and future directions in virome research. In addition, virome profiling remains technically and analytically challenging. Low viral biomass, dominance of bacterial sequences, and variability in sample processing can introduce pre-analytical bias (Gregory et al., 2020; Wang et al., 2023). The absence of a universal phylogenetic marker for viruses necessitates large-scale metagenomic approaches, increasing cost and complexity (Garmaeva et al., 2019). Moreover, incomplete reference databases, ongoing taxonomic revisions, and methodological variability hinder accurate annotation and comparative analyses (Eisenhofer et al., 2019; Sandybayev et al., 2022; Roux et al., 2015; Santiago-Rodriguez et al., 2022). These limitations collectively underscore the difficulties facing the virome field.

## 2 Interaction among the gut virome, gut bacteriome, and host immunity across the lifespan

Growing evidence highlights the complex interplay among the human gut virome, gut bacteriome, and host immunity, particularly during aging, underscoring its potential role in regulating immune responses and maintaining homeostasis, both essential for healthy longevity.

**FIGURE 2:**
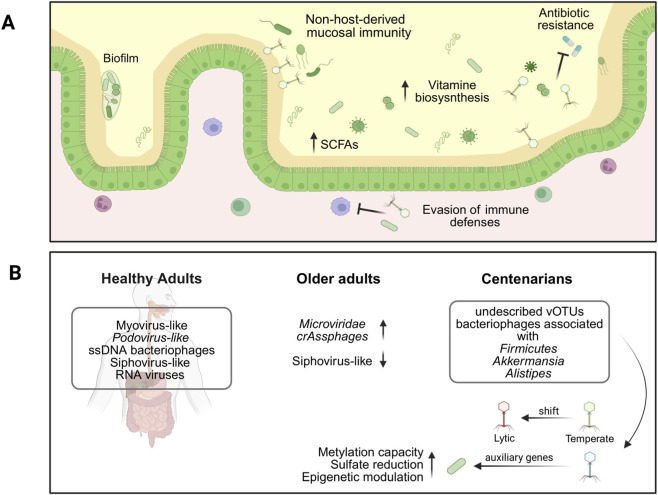
Physiological functions of the gut virome in healthy adults and their changes during aging. **(A)** In healthy adults, the gut virome contributes to host homeostasis by shaping bacterial community structure, enhancing biofilm formation, promoting vitamin and amino acid biosynthesis, supporting short-chain fatty acid (SCFA) production, and modulating immune responses. **(B)** During aging, the gut virome undergoes compositional shifts whose functional consequences remain largely unexplored. In centenarians, the gut virome exhibits distinctive features, including increased lytic activity and a high prevalence of phage-encoded auxiliary metabolic genes involved in sulfur metabolism. These traits may contribute to microbial stability, immune modulation, and healthy longevity.

Gut bacteriophages, estimated to number over 10^15^ in the human microbiota, are increasingly recognized as potential modulators of microbial dynamics and host immunity (Mahmud et al., 2024). Their life cycles follow distinct patterns: (i) lytic phages, which destroy bacterial hosts (*e.g.*, T4 phage); (ii) lysogenic or temperate phages, which integrate into bacterial genomes and can switch to a lytic cycle under certain conditions (*e.g.*, λ phage); and (iii) pseudolysogenic phages, which replicate and release progeny without lysing the host (*e.g.*, M13). Beyond shaping bacterial populations, phages may facilitate bacterial colonization through phage-encoded proteins that enhance adhesion, biofilm formation, antibiotic resistance, and evasion of host immune defenses (Torres-Barcelo, 2018; Popescu et al., 2021; Wang et al., 2025). Accumulation of phages in the mucosal layer can also serve as a non-host-derived protective barrier against pathogenic bacterial invasion (Popescu et al., 2021). Indeed, the gut virome harbors a diverse population of commensal phages that closely interact with the intestinal mucosa. Adherent phages can reach densities of up to 10^9^ per biopsy, supported by specific adaptations such as immunoglobulin-like domains, found in phages infecting *Escherichia coli*, like T4, that bind to mucins and epithelial glycoproteins. The presence of similar domains across various phage families suggests a conserved mechanism for mucosal association (Popescu et al., 2021). Through the remodeling of bacterial communities and modulation of microbial metabolism, phages can indirectly influence immune development and function. For instance, by selectively targeting bacteria involved in the production of short-chain fatty acids, vitamin biosynthesis, or amino acid metabolism, phages may alter the availability of key microbial-derived metabolites that support epithelial integrity and immune regulation (Leshem et al., 2020). Temperate phages can further influence bacterial metabolism through horizontal gene transfer, while phage-mediated lysis contributes to nutrient recycling within the gut ecosystem (Devoto et al., 2019; Borodovich et al., 2022) (Figure 2). Evidence from pediatric cohorts supports the notion that balanced bacteriophage-bacterial interactions are also essential for host developmental trajectories (Desai et al., 2020). The immunomodulatory potential of the gut virome has also been demonstrated in experimental models. Ritz et al. (2024) showed that transplantation of the fecal virome mitigated stress-induced behavioral changes and restored immune cell populations, cytokine profiles, and gene expression in the amygdala of mice.

In healthy adults, the gut virome is mainly composed of double-stranded DNA tailed phages from the order *Caudoviricetes*, along with single-stranded DNA bacteriophages and RNA viruses (Vitetta et al., 2018; Tan et al., 2021). *Caudoviricetes*, which represent a core component of the intestinal viral community, is a highly diverse class that groups all tailed bacterial and archaeal viruses with icosahedral capsids and double-stranded DNA genomes. It was recently redefined by the ICTV, which dissolved the order *Caudovirales* and the families *Myoviridae*, *Podoviridae* and *Siphoviridae*. In accordance with this classification, in this review we use the terms “Myovirus-like”, “Siphovirus-like” and “Podovirus-like” morphology/morphotype only as morphological descriptors of tail structure and for historical reference, without implying any formal taxonomic rank. While the gut virome composition remains relatively stable in healthy adults, recent studies have begun to uncover how it changes throughout the human lifespan. Gregory and colleagues (Gregory et al., 2020), through the construction of a comprehensive gut virome database from 1,986 individuals across 16 countries, were able to trace age-associated variations in healthy Western cohorts ranging from 0 to 3 years to over 65 years of age. They found a progressive increase in overall viral richness from infancy to adulthood, followed by a significant decrease in older adults. However, not all viral families followed the same pattern. For instance, while Siphovirus-like morphology mirrored this trajectory, *Microviridae* showed a steady and continuous increase from childhood throughout the lifespan. A similar age-related trend was observed for crAssphages, likely due to the progressive expansion of their ecological niches within the gut virome. Accordingly, Mayneris-Perxachs et al. (2022) reported that, with increasing age, members of the Siphovirus-like morphology family tend to decline, whereas *Microviridae* become progressively more abundant. Despite high interindividual variability, temperate phages within *Caudoviricetes* and *Microviridae* remain dominant in the gut of older adults. Supporting these observations, Johansen et al. (2023) found that virome diversity and composition are closely linked to the bacteriome, and their findings also revealed a marked increase in *Crassvirales* abundance and prevalence in individuals over 60 —a pattern that persists into advanced old age (Table 1). Thus far, investigations into alterations in virome composition during aging have been primarily descriptive, with limited exploration of their functional implications both locally within the gut and systemically. Addressing this gap will be essential to uncover potential mechanistic links with age-related pathologies and to identify novel virome-based therapeutic targets, as well as interventions aimed at extending healthspan.

**TABLE 1:** Representative components of the gut virome in older adults.

Family	Genome	Main viral species or genera infecting humans	Main hosts	References
*Adenoviridae*	dsDNA	HAdV-A, HAdV-B, HAdV-C, HAdV-D, HAdV-E, HAdV-F, HAdV-G	Humans, other mammals, birds	Yinda et al. (2019), Dhingra et al. (2019), Guo et al. (2017)
*α-, β-* and *γ- Herpesviridae*	dsDNA	HHV-1, HHV-2, HHV-3, HHV-4, HHV-5, VZV, HHV-6A, HHV-6B, HHV-7, HHV-8, CMV, EBV	Humans and other mammals	James et al. (2024), Yinda et al. (2019), Lu et al. (2021)
*Anelloviridae*	+ssDNA	20 human *Alphatorquevirus* (TTV)	Humans, non-human primates	Yinda et al. (2019), Gregory et al. (2020), Guo et al. (2017), Kaczorowska et al. (2022)
*Anelloviridae*	+ssDNA	38 *Betatorquevirus* (TTMV)	Humans	Yinda et al. (2019), Gregory et al. (2020), Guo et al. (2017), Kaczorowska et al. (2022)
*Anelloviridae*	+ssDNA	15 *Gammatorquevirus (*TTMDV)	Humans	Yinda et al. (2019), Gregory et al. (2020), Guo et al. (2017), Kaczorowska et al. (2022)
*Asfarviridae*	dsDNA	ASFV	Pigs	Yinda et al. (2019), Guo et al. (2017)
*Astroviridae*	+ssRNA	HAstV	Humans, other mammals, birds	Yinda et al. (2019), Guo et al. (2017)
*Autographiviridae*	dsDNA	*Escherichia* phage T7 *Salmonella* phage SP6 *Pseudomonas* phage phiKMV *Klebsiella* phage KP34 *Pseudomonas* phage LUZ19 *Yersinia* phage φR1-37	Bacteria	Zhang et al. (2025)
*Baculoviridae*	dsDNA	-	Insect larvae (primarily Lepidoptera, hymenoptera and Diptera)	Guo et al. (2017)
*Caliciviridae*	+ssRNA	*Norovirus* and *Sapovirus*	Humans, other mammals, birds, fish and amphibians	Yinda et al. (2019)
*Circoviridae*	ssDNA	HCirV may cause hepatitis in humans*	Mammals and birds	Liang and Bushman (2021), Yinda et al. (2019), Gregory et al. (2020), Guo et al. (2017), Wu et al. (2024)
*Coronaviridae*	+ssRNA	*Alphacoronavirus* and *Betacoronavirus*	Humans, other mammals, birds	Guo et al. (2017)
CrAss-like viruses	dsDNA	*crAss-like phage*	*Bacteroides* spp.	Zhang et al. (2025), Gregory et al. (2020), Lu et al. (2021)
*Flandersviridae*	dsDNA	Bacteriophages	*Bacteroides* spp.	Zhang et al. (2025)
*Flaviviridae*	+ssRNA	Dengue virus, zika virus, HCV, tick-borne encephalitis virus	Humans, other mammals, birds, reptiles, amphibians	Guo et al. (2017)
*Gratiaviridae*	dsDNA	Emerging taxonomic group	*Bacteroides* spp.	Zhang et al. (2025)
*Herelleviridae*	dsDNA	Bacteriophages	*Firmicutes* spp.	Zhang et al. (2025), Lu et al. (2021)
*Inoviridae*	ssDNA	*Escherichia* virus M13 *Pseudomonas* virus Pf1	Gram-negative bacteria	James et al. (2024), Zhang et al. (2025), Chaudhari et al. (2023), Gregory et al. (2020)
*Microviridae*	ssDNA	Bacteriophages	*Escherichia coli* and related *Enterobacteriaceae*	Zhang et al. (2025), Johansen et al. (2023), Mayneris-Perxachs et al. (2022), Gregory et al. (2020), Lu et al. (2021), Guo et al. (2017), Zhanbo et al. (2024)
Myovirus-like morphology	dsDNA	Bacteriophages	*Escherichia coli*, *Pseudomonas* spp., *Bacillus* spp.	James et al. (2024), Johansen et al. (2023), Zhang et al. (2025), Chaudhari et al. (2023), Vitetta et al. (2018), Gregory et al. (2020), Lu et al. (2021)
*Papillomaviridae*	dsDNA	HPV16, HPV18	Humans and other mammals	Zhanbo et al. (2024)
*Picobirnaviridae*	dsRNA	Human picobirnavirus genogroup I, human picobirnavirus genogroup II (uncertain pathogenicity in gastroenteritis cases)	Humans and other mammals	Yinda et al. (2019)
*Picornaviridae*	+ssRNA	Rhinovirus, enterovirus, hepatovirus Main species which change with age: Eduovirus, teseptimavirus and lubbockvirus	Humans, other mammals, birds	Zhang et al. (2025), Yinda et al. (2019), Guo et al. (2017)
Podovirus-like morphology	dsDNA	Bacteriophages	Gram-negative bacteria	James et al. (2024), Johansen et al. (2023), Zhang et al. (2025), Chaudhari et al. (2023), Vitetta et al. (2018), Gregory et al. (2020), Lu et al. (2021)
*Polyomaviridae*	dsDNA	BK virus, JC virus, MCPyV	Humans, other mammals and birds	Guo et al. (2017)
*Poxviridae*	dsDNA	Variola virus, Mpox, Molluscum tanapox virus	Humans, other mammals, birds, reptiles	Guo et al. (2017)
*Quimbyviridae*	dsDNA	Bacteriophages	Bacteroidetes	Zhang et al. (2025)
*Reoviridae*	dsRNA	*Rotavirus* A and PRV	Humans, other mammals, birds, insects, fish and plants	Guo et al. (2017)
*Retroviridae*	+ssRNA	HERVs, HIV-1, HIV-2, HTLV-1, HTLV-2	Humans, other mammals, birds, and reptiles	James et al. (2024), Yinda et al. (2019)
Siphovirus-like morphology	dsDNA	Bacteriophages	Bacteria and archaea	James et al. (2024), Johansen et al. (2023), Zhang et al. (2025), Chaudhari et al. (2023), Mayneris-Perxachs et al. (2022), Vitetta et al. (2018), Gregory et al. (2020), Lu et al. (2021), Zhanbo et al. (2024)
*Tectiviridae*	dsDNA	Bacteriophages	Gram-negative bacteria	James et al. (2024), Lu et al. (2021)
*Togaviridae*	+ssRNA	Alphavirus and rubivirus	Humans, other mammals, birds	Guo et al. (2017)

HAdV, human mastadenovirus; HHV, human herpesvirus; CMV, cytomegalovirus; EBV, Epstein–Barr Virus; TTV, torque teno virus; TTMV, torque teno minivirus; TTMDV, torque teno midivirus; ASFV, African sSwine fFever vVirus; HAstV, human astrovirus; HCirV, human circovirus; HCV, Hepatitis C virus; HPV, human papillomavirus; BK, virus, Poliomavirus BK; JC, virus, John Cunningham virus; MCPyV, merkel cell polyomavirus; PRV, pseudorabies virus; HERV, human endogenous retrovirus; k HIV-1; HIV-2, human immunodeficiency virus; HTLV-1/2, Human T-lymphotropic virus.

A distinct and noteworthy feature of the aging microbiota is the gut virome of centenarians, a highly individualized, metabolically integrated, and functionally active viral community that offers valuable insights into the biology of healthy aging and exceptional longevity. High-resolution metagenomics has revealed that centenarians harbor an unusually rich and diverse virome, markedly distinct from that of younger and even older adults under the age of 100 (Johansen et al., 2023). Their virome includes many previously undescribed viral Operational Taxonomic Units (vOTUs), over 1,700 without matches to known viral genome, predominantly bacteriophages associated with *Firmicutes*, including *Clostridium* species such as *C. scindens*, *C. innocuum*, and *C. symbiosum*, as well as genera like *Akkermansia* and *Alistipes*. The expansion of viral genera found almost exclusively in centenarians suggests long-standing phage–bacteria associations shaped by decades of co-evolution and microbial stability.

A defining characteristic of this virome is a shift toward increased lytic activity, reflected in higher viral-to-bacterial ratios and a reduced prevalence of temperate phages. This pattern may be linked to the chronic low-grade inflammation typical of aging, which can promote prophage induction (Zhang et al., 2025). Another distinctive element is the enrichment of phage-encoded auxiliary metabolic genes (AMGs), particularly those involved in sulfur metabolism, such as S-adenosylmethionine synthetase, phosphoadenosine-phosphosulfate reductase, and DNA cytosine methyltransferase, which substantially contribute to the metabolic potential of the centenarian gut (Figure 2).

Although current data are primarily descriptive, established ecological and host–microbe principles allow functional inferences. High phage diversity is known to stabilize microbial communities by preventing overgrowth of fast-growing taxa and promoting long-term ecological balance (Godsil et al., 2025), mechanisms consistent with the distinctive microbial configuration and resilience repeatedly observed in centenarians (Lozada-Martinez et al., 2024). The AMGs enriched in their virome are associated with redox homeostasis, detoxification pathways, and genomic stability, suggesting that phage-encoded metabolic functions may enhance microbial robustness and support beneficial host–microbe interactions (Shackelford et al., 2021; Gao et al., 2026). Additional protective effects may arise from phage interactions with SCFA producers. By modulating the turnover and metabolic activity of these bacteria, phages could indirectly sustain SCFA-mediated functions, including epithelial barrier reinforcement, immune tolerance, and control of low-grade inflammation (Kim, 2023; Ahmad et al., 2025).

## 3 Factors affecting virome composition

### 3.1 Diet and geographical location

Diet can influence the virome indirectly, by modulating the bacteriome and thereby altering phage composition, and directly, by activating or inhibiting bacteriophage functions (Howard et al., 2024). In a mouse model of obesity, a high-fat/high-sucrose diet enriched temperate *Caudoviricetes* phages in the mucosa compared to the lumen and eliminated spatial distinctions between compartments (Kim and Jin, 2016). A high-fat diet alone reduced Siphovirus-like morphotype and increased *Microviridae*, with viral communities showing greater virulence toward bacterial hosts (Schulfer et al., 2020). Among direct dietary effects, prophage modulation is particularly relevant. Fructose metabolism by *Lactobacilli* produces acetic acid, which triggers prophage activation (Mahmoud et al., 2025). Boling and colleagues (Boling et al., 2020) screened common foods and identified several prophages inducers in *Bacteroides thetaiotaomicron* and *Enterococcus faecalis*, including aspartame, stevia, and bee propolis. Conversely, certain substances suppress prophage activation (*e.g.*, cinnamon, oregano, and specific flavonoids) or reduce phage infectivity (*e.g.*, tea extracts, cranberry juice, and ascorbic acid) (Marongiu et al., 2021). At the population level, virome variation reflects region-specific diets and other environmental conditions associated with geographical location, including urbanization-related lifestyle factors. In a large Chinese cohort, geography was the primary driver of gut DNA virome composition, exerting a stronger influence than the bacterial microbiome; urban residents showed reduced virome diversity, potentially increasing susceptibility to chronic inflammatory diseases (Zuo et al., 2020). Much of this effect is attributed to population-specific dietary patterns, which, together with host factors such as age, sex, and ethnicity, shape distinct viral signatures. Comparative studies confirm that viral species correlate with regionally defined diets, subsistence strategies, and lifestyles, with hunter-gatherer displaying higher virome diversity than urban populations (Rampelli et al., 2017). Population-specific viral markers have also been identified: Torque teno midi virus (TTMDV) 2 and HHV7 in the Hadza, *Betapapillomavirus* NC015692.1 and TTMDV2 in the Matses, Human papillomavirus type 178 in Italians, and HHV 8 in the US population. A dedicated mention is warranted for probiotics, which can be integrated into the daily diet. *Lactobacillus* and *Bifidobacteria* are among the most abundant and well-studied genera. They counteract pathobionts colonization through antibiotic-like compounds, ecological competition, immune stimulation, and reinforcement of intestinal mucosal integrity. By modulating bacterial communities, probiotic may also influence viral replication niches, phage–bacteria dynamics, and overall gut ecosystem homeostasis (see Vitetta et al., 2018 for review).

### 3.2 Genetics

Host genetics influence gut virome composition primarily through modulation of immune responses and barrier integrity. Polymorphisms in immunity-related genes can alter viral recognition and susceptibility (Govender and Ghai, 2025), while physical and chemical barriers, such as the intestinal mucosa, regulate viral access and colonization (Buhner et al., 2006; Moehle et al., 2006; Muise et al., 2009). Host-encoded restriction factors, including APOBEC3 cytidine deaminases, shape viral evolution and persistence in a tissue-specific manner; for example, APOBEC3B expression in the colon is linked to mutational biases in parvovirus B19 (Pyöriä et al., 2024). Genetic background also affects genomic stability and chromatin architecture, influencing viral genome integration even in non-cancerous tissues for EBV, HHV-6B, and Merkel cell polyomavirus (Pyöriä et al., 2024). Beyond these mechanisms, ethnicity and heritable factors broadly shape virome diversity and functional potential, as shown in comparisons between Chinese and Pakistani populations, where virome profiles also co-varied with bacterial microbiota (Yan et al., 2021).

### 3.3 Bacterial and viral co-colonization

Bacteriophages modulate bacterial communities through transposition, induction, and horizontal gene transfer (Keen and Dantas, 2018). Specific *Caudoviricetes* show positive associations with lactic acid bacteria and negative correlations with *Bacteroides* (Mayneris-Perxachs et al., 2022), whereas *Microviridae* display the opposite pattern. Age- and species-specific co-occurrence patterns were demonstrated in *Macaca fascicularis*, linking classical phage families (Myovirus-like morphology, Siphovirus-like morphology, Podovirus-like morphology) to core bacterial phyla across life stages (Tan et al., 2021). In disease contexts, enrichment of these phage groups correlates with dysbiosis-associated taxa in chronic kidney disease, implicating viral contributions to disease progression (Zhang et al., 2025).

### 3.4 Medications

Broad-spectrum antibiotics disrupt bacterial hosts and can induce temperate phages (Ubeda et al., 2005; Misson et al., 2023), while phage-encoded antibiotic resistance genes facilitate the spread of resistance within the microbiome (Pfeifer et al., 2021), increasing virulence and resistance gene load (Chopyk et al., 2023). Antiviral treatments such as antiretrovirals reduce target virus loads and reshape gut viral diversity (Bhagchandani et al., 2024; Liang and Bushman, 2021). Immunosuppressants promote reactivation of CMV, BK virus, EBV, HHV-6A, and alter levels of immune-sensitive commensal viruses such as *Anelloviridae* (Fujimoto et al., 2020; Giacconi et al., 2020; Rosiewicz et al., 2024). Chemotherapy exerts cytotoxic and immunosuppressive effects promoting the expansion of latent or opportunistic viruses (Henze et al., 2022; Opzoomer et al., 2019; Yang et al., 2024).

### 3.5 Smoking

The impact of smoking on the gut virome remains largely unexplored, but evidence from other mucosal sites underscores its relevance. Lung virome analyses show significant differences between smokers and nonsmokers: although 29% of viral populations were shared, several phage taxa varied markedly. *Prevotella* phages were more abundant in smokers, whereas *Lactobacillus* and *Gardnerella* phages were more prevalent in nonsmokers. Rare phage populations also showed group specificity (Gregory et al., 2018). Preliminary gut data indicate smoking-associated shifts in virome composition and functional potential, including altered abundance of vOTUs enriched in auxiliary metabolic genes (Istvan et al., 2024).

## 4 Mechanistic links between the virome and aging: a geroscience framework

Accumulating evidence suggests that age-related changes in the human virome are not merely descriptive, but reflect a set of interconnected mechanisms that converge on the hallmarks of aging. Within the Geroscience framework, four mechanistic domains appear particularly relevant.

### 4.1 Viral contribution to immune exhaustion and immunosenescence

Persistent viral antigens-derived from chronic herpesvirus infections, reactivated latent viruses, and lifelong exposure to commensal viruses such as *Anelloviridae*, contribute to chronic stimulation of innate and adaptive immunity. This sustained antigenic load drives expansion of terminally differentiated CD8^+^ T cells, contraction of the naïve T cell pool, and impaired antiviral responses (Rodriguez and Parra-Lopez, 2024; Hu et al., 2025; Mangiaterra et al., 2024). The progressive rise of TTV viremia with age (Giacconi et al., 2020; Giacconi et al., 2018) exemplifies how reduced immune control enables viral persistence and may serve as a readout of immune exhaustion as observed in other chronic infections (Eslami et al., 2025; Hu et al., 2025). These processes converge on core Geroscience mechanisms including loss of proteostasis, impaired intercellular communication, and dysregulated immunity (Mittelbrunn and Kroemer, 2021; Fortunato et al., 2025).

### 4.2 Prophage induction and chronic inflammation

Inflammaging creates a pro-oxidant, cytokine-rich environment that favors prophage induction in gut bacteria (Mahmoud et al., 2025). Prophages can naturally shape gut bacterial populations and, when activated, contribute to the loss of beneficial intestinal symbionts, thereby worsening dysbiosis (Nanda et al., 2015). Increased lytic activation releases bacterial cell-wall components, toxins, and immunostimulatory DNA, amplifying mucosal and systemic inflammation (Mahmoud et al., 2025). Prophage induction also results in the production of numerous phage particles capable of penetrating the intestinal epithelial layer (Nguyen et al., 2018). Once across the barrier, intestinal phages can directly interact with host immune cells, enhancing the production of chemokines and cytokines (Seo and Kweon, 2019; Federici et al., 2021). This self-reinforcing loop-chronic inflammation promoting prophage activity, which in turn fuels inflammation, links virome dynamics to age-associated barrier dysfunction, metabolic dysregulation, and immune remodeling.

### 4.3 Viral-bacterial metabolic interactions and host energy homeostasis

Phages modulate bacterial networks involved in essential metabolic pathways such as short-chain fatty acid (SCFA) production, sulfur metabolism, and vitamin biosynthesis (Jessen and Bui, 2025). Auxiliary metabolic genes encoded by bacteriophages, particularly those enriched in centenarians, can enhance microbial metabolic flexibility and resilience (Johansen et al., 2023). Conversely, virome-driven shifts in *Lactococcus* or SCFA-producing taxa may impair epithelial integrity, mitochondrial metabolism, and neuroimmune communication, (Tetz et al., 2018; Yadav et al., 2022), contributing to metabolic reprogramming a central mechanism of aging.

### 4.4 Persistent virome signatures as biomarkers of biological age

Distinct viral communities, most notably the circulating virome, mirror the functional state of the immune system and correlate with biological age, frailty, and mortality risk (Kananen et al., 2015; Giacconi et al., 2023; Cianfruglia et al., 2025). Viral reactivation patterns (*e.g.*, herpesvirus, human endogenous retroviruses (HERVs), torque teno virus, TTV) further track with epigenetic drift, cellular senescence, and loss of homeostatic regulation (Pantry and Medveczky, 2017; Noppert et al., 2020; Mao et al., 2024; Fortunato et al., 2025). These signatures support the concept that the virome is both a sensor and a potential driver of aging trajectories (Momkus et al., 2025; Fortunato et al., 2025; Johansen et al., 2023; Cianfruglia et al., 2025).

Together, these domains outline a mechanistic model wherein the virome interacts dynamically with the immune system, microbial metabolism, epithelial barriers, and systemic inflammation, contributing to the trajectory of aging and age-related disease. This conceptual synthesis integrates the gut and circulating virome into the Geroscience paradigm, highlighting both causative pathways and biomarker potential while underscoring the need for longitudinal mechanistic studies.

## 5 Emerging roles of the human virome in brain inflammation and neurodegeneration

Age-related alterations in the gut–brain axis are increasingly recognized for their role in pathological outcomes. Gut dysbiosis can compromise the integrity of the intestinal barrier (“leaky gut”), enabling microbial translocation and systemic inflammation. This inflammatory *milieu* may disrupt the blood–brain and the blood–cerebrospinal fluid barriers, allowing neurotoxic compounds to enter the central nervous system and promoting neuroinflammation, neuronal injury, and cognitive decline (Lai et al., 2025; Olejnik et al., 2025).

### 5.1 Metagenomic studies

**FIGURE 3:**
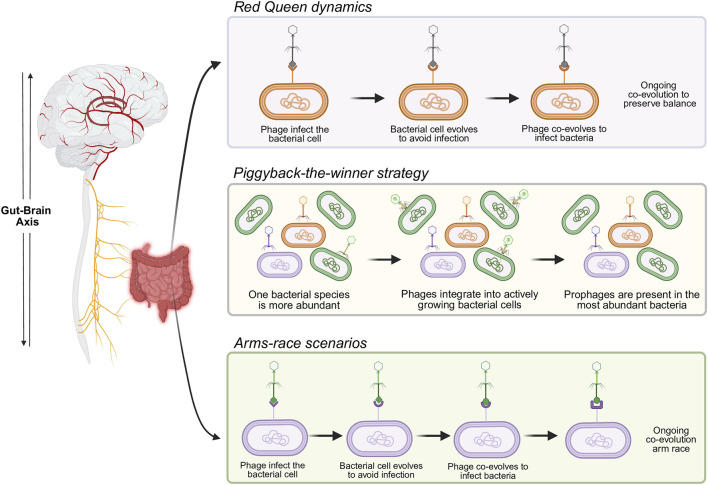
Diverse evolutionary dynamics between phages and bacteria. The interactions between phages and bacteria range from Red Queen dynamics, in which both phages and bacteria must continuously evolve to maintain equilibrium, to piggyback-the-winner strategies, where temperate phages integrate into actively growing bacterial cells without lysing them, and arms-race scenarios, characterized by an evolutionary escalation in which each side adapts to outcompete the other. Furthermore, even without lysing the host, some prophages can reduce bacterial fitness and alter phenotypes by encoding genes for toxins, virulence factors, or antibiotic resistance.

Although less studied than the bacteriome, the virome is increasingly implicated in Alzheimer’s disease (AD) and Parkinson’s disease (PD). In AD, metagenomics revealed reduced phage richness, with a 9% decrease in *Uroviricota* and Siphovirus-like morphology and an 11% increase in *Poxviridae*; several *Lactococcus* phages also declined, potentially affecting bacterial diversity and lactate metabolism (Ghorbani et al., 2023). In mild cognitive impairment (MCI), *Lactobacillus* phages Sha1 and LF1, *Klebsiella* virus KP36, and *Lactobacillus* phage blL309 were enriched compared to controls, while specific phages (*e.g.*, *Clostridium* phage vBC peS CP51, *Lactococcus* phages jm3 and ul36) showed moderate diagnostic accuracy (54%–58%) and were more abundant in individuals with more severe cognitive deficits (Chaudhari et al., 2023). Kazen et al. (2025) identified 28 phages exclusive to amnestic MCI due to AD and 8 exclusives to healthy controls, with abundance shifts linked to disruptions in viral-encoded metabolic pathways (ATP/ADP synthesis from inosine monophosphate, propionyl-CoA metabolism, and methanogenesis) and increased lysogenic phages. In PD, Bedarf et al. (2017) observed reduced bacteriophages and archaeal phages *versus* controls, with no differences in prophage and plasmid content; this was confirmed in a spouse-controlled cohort to reduce lifestyle confounding (Mao et al., 2021). Conversely, Duru et al. (2025) found reduced plasmid alpha diversity, significant beta diversity differences, enrichment of *Microviridae* and *Tectiviridae*, and increases in phages infecting *Bifidobacterium* and *Ruthenibacterium*. Classification models moderately distinguished PD from controls using plasmid profiles (AUC = 0.661) and improved using phage profiles (AUC = 0.746). Overall, phages remain the most influential virome component in shaping microbiota due to diverse bacterial interactions (Figure 3) (Tisza and Buck, 2021).

### 5.2 Mechanistic links

Beyond correlative associations, mechanistic links have been proposed. Declines or lifestyle-driven shifts in *Lactococcus* phages may alter *Lactococcus* populations and disrupt the balance between L- and D-lactic acid. L-lactate supports neuronal metabolism, memory, protein synthesis, synaptic remodeling, and axonal excitability, whereas D-lactate can impair neuronal uptake of L-lactate and hinder function (Ghorbani et al., 2023). In PD, a predominance of strictly lytic *Lactococcus* phages, despite stable overall abundance, was linked to a >10-fold reduction in *Lactococcus* species, potentially lowering microbiota-derived dopamine and increasing gut permeability. These changes may contribute to gastrointestinal symptoms, systemic inflammation, and the initiation of neurodegenerative processes (Tetz et al., 2018). Aggregates of α-synuclein in the enteric nervous system and evidence of gut-to-brain propagation via the vagus nerve further support a mechanistic bridge between virome-driven dysbiosis and central pathology (Braak et al., 2006; Kim et al., 2019).

### 5.3 Experimental models

Longitudinal monitoring in a murine AD model showed a simplified viral ecological network, stable alpha diversity, and increased beta diversity over time compared with wild-type controls. Virome composition shifted dynamically, with elevated *Lahndsivirus rarus* at 3 months and increased *Lactobacillus* prophages Lj771, KC5a, and phi Jlb1 at 6 months, paralleling disease progression (Zhang et al., 2024).

### 5.4 Human cohort studies

In older adults (60–69, 70–79, ≥80 years), progressive virome changes were linked to metabolic alterations, including increased activity in tricarboxylic acid cycle VI and NAD salvage pathway III, reduced pyruvate fermentation, and associations with cognitive status. Cognitively impaired individuals showed fewer viral species and distinct virome composition, with higher Myovirus-like morphology and Podovirus-like morphology, lower Siphovirus-like morphology and *Inoviridae*, and presence of *Tectiviridae* (absent in controls). They also exhibited altered phage–bacteria–metabolism interactions, notably reduced anaerobic sucrose degradation consistent with depletion of carbohydrate-metabolizing bacteria, potentially contributing to neuroinflammation (James et al., 2024).

## 6 The circulating virome in aging and age-related diseases

The circulating virome, comprising the full spectrum of viruses present in the bloodstream, is emerging as a key biomarker of aging and immune function. Among its major components are the *Anelloviridae* family, comprising three major human-infecting genera: *Alphatorquevirus* (torque teno virus, TTV), *Betatorquevirus* (torque teno minivirus, TTMV), and *Gammatorquevirus* (torque teno midivirus). These viruses possess small, circular single-stranded DNA genomes and establish lifelong asymptomatic infections. In 2021, Varsani and colleagues, proposed a major taxonomic revision of the family, expanding the number of genera from 14 to 30 and introducing new demarcation criteria. Following this update, in 2023 the International Committee on Taxonomy of Viruses (ICTV) adopted a binomial nomenclature system at the species level (Varsani et al., 2023) formally named *Alphatorquevirus homin*, *Betatorquevirus homini*. In total, 30 genera and 155 species are now recognized, including newly human-infecting genera such as *Hetorquevirus*, *Yodtorquevirus*, *Lamedtorquevirus*, *Memtorquevirus*, *Samektorquevirus*, and *Sadetorquevirus* (Laubscher et al., 2023; Laubscher et al., 2024). Recent metagenomic studies confirmed the presence of these genera in multiple human cohorts. Do et al. (2024) identified seven novel anelloviruses in cervicovaginal lavage fluid from women with HIV, expanding known ecological niches. This study provided the first evidence that these lineages can infect humans outside the bloodstream, thereby expanding the ecological niches known for anelloviruses. Modha et al. (2025) retrieved 829 new genomes from public microbiome datasets. While the majority clustered within *Alphatorquevirus*, *Betatorquevirus*, and *Gammatorquevirus*, the authors also reconstructed genomes of *Hetorquevirus* as well as multiple representatives of *Memtorquevirus* and *Samektorquevirus*, thereby substantially broadening the genomic diversity recognized in humans. Finally, Phumiphanjarphak et al. (2025) analysed over one thousand Thai whole-genome sequencing datasets, uncovering 511 anellovirus genomes. This large-scale population study recovered complete genomes of *Alphatorquevirus*, *Betatorquevirus*, *Gammatorquevirus*, and notably *Hetorquevirus*, while additional evidence of *Lamedtorquevirus*, *Samektorquevirus*, and *Yodtorquevirus* was detected at lower genomic resolution. Together, these studies confirm that recently defined genera represent genuine human virome components.

Current evidence supports a model in which circulating anelloviruses primarily reflect host immune competence rather than acting as direct pathogenic drivers. Most associations reported to date are observational and context-dependent, consistent with a role for anelloviruses as sensitive indicators of immune regulation across the lifespan. Nevertheless, emerging links with immune remodelling, epigenetic reprogramming, inflammatory biomarkers, and vaccine responsiveness suggest that persistent anellovirus–host interactions may contribute indirectly to age-related immune phenotypes. These observations underscore the need for longitudinal and mechanistic studies to disentangle correlation from causation in virome–aging relationships.

Long-term studies by Kaczorowska et al. (2022), Kaczorowska et al. (2023) tracking two healthy individuals over more than 30 years. In both subjects TTV was the dominant and most persistent genus, and TTV viremia increased moderately over time, likely reflecting age-related immune decline. Importantly, each individual maintained a distinct and stable core anellovirome, representing a personal virome signature. Large-scale sequencing studies support the idea of a “personal anellovirome,” showing that individuals carry unique and stable anellovirus lineages, seldom shared across unrelated people and modifiable by exposures such as transfusions (Arze et al., 2021). Moreover, metagenomic surveys indicate that anelloviruses may represent up to 97% of plasma viral reads (Cebria-Mendoza et al., 2021). Consistently, Cordey et al. (2021) found *Anelloviridae* in almost all of 816 febrile children in Tanzania, with 99% showing co-infections of the three classical genera and hundreds of genotypes within single individuals, underscoring their ubiquity and remarkable intra-host diversity early in life. In contrast, Ma et al. (2024) profiled the plasma virome of over 1,300 hospitalized patients, including many with hematologic disorders. *Herpesviridae*, *Anelloviridae*, and *Flaviviridae*, along with fungal and bacterial DNA, were among the most common taxa detected. Patients with hematologic conditions, especially non-neutropenic ones, exhibited significantly higher viral richness and TTV prevalence. TTV load strongly correlated with immune markers such as C-Reactive Protein and absolute neutrophil count, and often co-occurred with *Herpesviridae* and *Flaviviridae*, particularly post-transplant, reinforcing its role as a marker of immune dysregulation. Luciola Zanette et al. (2023) reported that patients with prostate cancer show enrichment of TTV, particularly genotypes 15, 16, and 22, and Human Pegivirus 1 compared with healthy donors, with distinct virome clustering suggesting that alterations in the circulating virome may reflect immune perturbations linked to aging and disease. Consistently, elevated TTV viremia has been associated with ischemic heart disease (Giacconi et al., 2024) physical frailty (Giacconi et al., 2023), age-related immunosenescence patterns (Giacconi et al., 2020), and increased all-cause mortality in older adults (Giacconi et al., 2018) and geriatric patients (Cianfruglia et al., 2025). TTV has also been identified as a potential prognostic biomarker in lung infections among immunocompromised patients (Zhu et al., 2024).

Overall, these studies show that the aging blood virome evolves with the immune system. TTV viremia rises with age in both healthy individuals and patients, indicating reduced immune control over commensal viruses. The long-term persistence of a core anellovirome (Kaczorowska et al., 2022) suggests that some viral lineages are tolerated and may have immunomodulatory roles. The circulating virome therefore represents a promising non-invasive biomarker of biological aging, immunosenescence, and age-related disease risk. Although its clinical significance is still being defined, virome profiling could enhance immunological and geriatric assessments.

Specifically, a prospective metagenomic study by Spezia et al. (2023) identified high viral loads of TTV-7 in some patients with Kawasaki disease.

**FIGURE 4:**
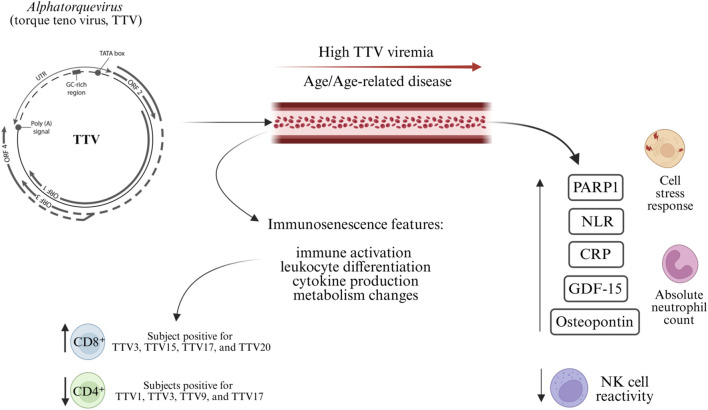
Anelloviridae as biomarkers of immune aging. The circulating virome, largely dominated by *Anelloviridae*, particularly *Alphatorqueviru*s (torque teno virus, TTV), reflects host immune status across the lifespan. A stable and personalized core anellovirome is maintained in healthy individuals, whereas aging and immune decline are associated with increased TTV load and species diversity. Elevated TTV viremia is associated with increased systemic inflammation, epigenetic remodelling, and a higher risk of age-related diseases, as well as with impaired natural killer (NK) cell activity and T-cell dysregulation, characterized by an imbalance between CD4^+^ and CD8^+^ T-cell subsets. Notably, distinct TTV species have been associated with increased CD8^+^ and reduced CD4^+^ T-cell counts.

Recent data from Novazzi et al. (2025) revealed that TTV load and species diversity increase with advancing age and progressive immune impairment. Species such as TTV1, TTV3, TTV5, TTV15, TTV19, TTV20, TTV21, TTV24, and TTV29, were enriched in older adults displaying features of immunosenescence. Several TTV species were associated with altered T-cell profiles, with reduced CD4^+^ percentages (TTV1, TTV3, TTV9, TTV17) and increased CD8^+^ cells (TTV3, TTV15, TTV17, TTV20). Moreover, higher species richness correlated with elevated CMV IgG titers, and increased PARP-1 expression and enzymatic activity, suggesting a link between virome diversification, inflammation, and cellular stress responses. Fortunato et al. (2025) uncovered a distinct DNA methylation pattern associated with TTV load in hospitalized older adults, highlighting pathways related to immune activation, leukocyte differentiation, cytokine production, and lipid metabolism, thereby revealing a potential epigenetic interface between viral persistence and immunosenescence (Figure 4). An overview of the main studies investigating the circulating human virome in older adults is provided in Table 2.

**TABLE 2:** Human blood virome in older adults.

Family	Genome	Main species detected	References
*Adenoviridae*	dsDNA	Mastadenovirus	Moustafa et al. (2017), Luciola Zanette et al. (2023), Autio et al. (2022)
*Anelloviridae*	+ssDNA	20 *Alphatorquevirus* (TTV)	Moustafa et al. (2017), Kaczorowska et al. (2022), Kaczorowska et al. (2023), Ma et al. (2024), Luciola Zanette et al. (2023), Autio et al. (2022), Cinti et al. (2025)
*Anelloviridae*	+ssDNA	38 *Betatorquevirus* (TTMV)	Moustafa et al. (2017), Kaczorowska et al. (2022), Kaczorowska et al. (2023), Ma et al. (2024), Luciola Zanette et al. (2023), Cinti et al. (2025)
*Anelloviridae*	+ssDNA	15 *Gammatorquevirus* (TTMDV)	Moustafa et al. (2017), Kaczorowska et al. (2022), Kaczorowska et al. (2023), Ma et al. (2024), Luciola Zanette et al. (2023), Cinti et al. (2025)
*Anelloviridae*	+ssDNA	5 human *Hetotorquevirus* *1* human *Lamedtorquevirus* *3* human *Memtorquevirus* *2* human *Sadetorquevirus* *4* human *Samektorquevirus* *2* human yod*torquevirus*	Laubscher et al. (2024), Laubscher et al. (2023), Modha et al. (2025), Phumiphanjarphak et al. (2025), Cordey et al. (2021)
*Flaviviridae*	+ssRNA	HGV; *Pegivirus columbiaense*; HCV	Moustafa et al. (2017), Ma et al. (2024), Luciola Zanette et al. (2023)
*Hepadnaviridae*	Partially dsDNA	HBV	Moustafa et al. (2017)
*Herpesviridae*	dsDNA	HSV-1, HHV-4, HHV-6A, HHV-6B, EBV, CMV, HHV-7, VZV, KSHV	Moustafa et al. (2017), Ma et al. (2024), Luciola Zanette et al. (2023), Autio et al. (2022)
*Orthomyxoviridae*	−ssRNA	Influenza A virus	Moustafa et al. (2017)
*Papillomaviridae*	dsDNA	HPV, betapapillomavirus 1, betapapillomavirus 4, gammapapillomavirus 1, gammapapillomavirus 9	Moustafa et al. (2017), Autio et al. (2022)
*Parvoviridae*	ssDNA	Human parvovirus B19	Moustafa et al. (2017), Ma et al. (2024)
*Polyomaviridae*	dsDNA	Merkel cell polyomavirus, trichodysplasia spinulosa polyomavirus; JCV	Moustafa et al. (2017), Ma et al. (2024)
*Retroviridae*	+ssRNA	HTLV-1/2; HIV-1/2	Moustafa et al. (2017)

TTV, torque teno virus; TTMV, Torque teno mini-virus; TTMDV, torque teno midi virus; HGV, hepatitis G virus; HCV, hepatitis C virus; HBV, Hepatitis B virus; HSV-1, Herpes simplex virus type 1; HHV, human herpesvirus; EBV, Epstein-Barr virus; CMV, cytomegalovirus; VZV, Varicella-Zoster virus; KSHV, Kaposi’s sarcoma-associated herpesvirus; HPV, papillomavirus; JCV, john cunningham virus; HTLV-1/2, Human T-lymphotropic virus; HIV, human immunodeficiency virus.

Table 3 summarizes the known clinical associations of circulating *Anelloviridae*. However, despite increasing evidence linking anelloviruses to immunosenescence and age-related diseases, our knowledge of the anellovirome in ageing remains incomplete, and its clinical applicability has yet to be established. Future longitudinal research is needed to clarify causality and assess if interventions targeting the virome can improve health in older adults.

**TABLE 3:** Circulating Anelloviridae: taxonomy and clinical interpretation.

ICTV genus/virus	Human evidence	Clinical interpretation	Key references
*Alphatorquevirus homin* (TTV)	Ubiquitous, lifelong viremia; dominant component of blood virome; strongly regulated by immune status; increases with immunosuppression, aging, frailty, ischemic heart disease, mortality and poor vaccine response, reflects systemic immune and inflammatory state	Robust biomarker of immune competence/immunosenescence (not proven causal agent)	Varsani et al. (2023), Ma et al. (2024), Luciola Zanette et al. (2023), Arze et al. (2021) , Kaczorowska et al. (2022), Kaczorowska et al. (2023) , Ma et al. (2024), Giacconi et al. (2018) , Giacconi et al. (2020) , Giacconi et al. (2023) , Giacconi et al. (2024) , Cianfruglia et al. (202) , Fortunato et al. (2025) , Zhu et al. (2024) , Cinti et al. (2025)
Exploratory studies on TTV species	Enrichment of specific TTV species in defined clinical contexts (*e.g.*, TTV-7 in Kawasaki disease; TTV-15, TTV-16 and TTV-22 in prostate cancer; multiple TTV species enriched in older adults with immunosenescence)	Hypothesis-generating associations only; no validated diagnostic or prognostic role; likely reflect host immune *milieu* rather than species-specific pathogenicity	Spezia et al. (2023) , Luciola Zanette et al. (2023) , Novazzi et al. (2025)
*Betatorquevirus homini* (TTMV)	Frequently co-detected with TTV; moderate viral loads; positively correlated with TTV; higher loads in vaccine non-responders	Emerging immune biomarker; may act as surrogate marker when TTV is undetectable	Arze et al. (2021) , Moustafa et al. (2017) , Kaczorowska et al. (2022), Kaczorowska et al. (2023) , Luciola Zanette et al. (2023) , Cinti et al. (2025)
*Gammatorquevirus homini* (TTMDV)	High prevalence; lower viral loads; correlated with other anelloviruses but not discriminative for vaccine response	Limited standalone clinical value; may contribute to total anellovirus burden	Arze et al. (2021) , Luciola Zanette et al. (2023) , Cinti et al. (2025)
Newly defined human genera (*Hetorquevirus*, *Lamedtorquevirus*, *Samektorquevirus*, *Yodtorquevirus*, etc.)	Detected by metagenomics in blood and extra-blood compartments; components of human virome	No established clinical relevance; important for taxonomy-aware interpretation	Varsani et al. (2023) , Laubscher et al. (2023) , Laubscher et al. (2024) , Do et al. (2024) , Modha et al. (2025), Phumiphanjarphak et al. (2025)

## 7 Viral pathobionts in aging: latency, reactivation and immune impact

The concept of viral pathobionts has gained prominence in the context of aging. Unlike obligate pathogens, these viruses persist asymptomatically in a delicate equilibrium with the host that can be disrupted by immunosenescence, chronic inflammation, microbiome dysbiosis, metabolic imbalance, chronic disease, or immune suppression, leading to viral reactivation and pathogenicity (Jochum and Steche, 2020).

Two major classes of viral pathobionts implicated in aging are human endogenous retroviruses (HERVs), and herpesviruses. HERVs are ancient viral elements integrated into the human genome that, despite lacking replicative capacity, can modulate host gene expression, particularly near immune-related loci (Belshaw et al., 2004). Their transcripts and proteins interact bidirectionally with the immune system, exerting both immunostimulatory and immunoregulatory effects (Grandi and Tramontano, 2018). HERVs have been implicated in various inflammatory, autoimmune, and neurological diseases; notably, the multiple sclerosis (MS)-associated retrovirus MSRV/HERV-W is actively expressed in the human brain and significantly upregulated in MS patients (Mameli et al., 2007). In AD, locus-specific upregulation of HERVs, particularly of the HERV-K superfamily, has been detected in brain tissues, often near genes implicated in neurodegeneration and inflammation. This expression positively correlates with interferon signalling and Toll-like receptor 8 (TLR8) activation, both hallmarks of chronic neuroinflammation, suggesting that derepressed HERVs may act as pro-inflammatory drivers in AD pathogenesis (Dawson et al., 2023). More recently, senescence-associated ERVs (SA-ERVs) have been shown to reactivate during cellular aging as a consequence of epigenetic deregulation, producing double-stranded RNA that activates innate immune pathways and sustains chronic type I interferon responses in aged tissues (Mao et al., 2024).

Among herpesviruses (order *Herpesvirales*, family *Herpesviridae*), EBV, CMV, and varicella-zoster virus (VZV) are most strongly linked to age-related diseases. Indeed, their reactivation, often triggered by immunosenescence or stress, contributes to inflammaging and further immune dysregulation (Suzich and Cliffe, 2018; Noronha et al., 2021; Gadoth et al., 2024).

### 7.1 Cytomegalovirus (CMV)

CMV is an ubiquitous β-herpesvirus that elicits strong, lifelong T-cell responses characterized by age-dependent memory inflation and immune evasion (Jergovic et al., 2019).

CMV is considered a major driver of immunosenescence, inducing clonal expansion of highly differentiated, senescent CD8^+^ T cells at the expense of the naïve T-cell pool, thereby reducing immune repertoire diversity (Rodriguez and Parra-Lopez, 2024). These CD8^+^ subsets express senescence markers (CD57, KLRG1) and display diminished effector capacity (Pickering et al., 2025). Longitudinal studies show that CMV-seropositive older adults frequently exhibit the immune risk profile, marked by low CD4/CD8 ratio, expanded CD8^+^ CD28^-^ T cells, and chronic inflammation, correlating with frailty, poor vaccine responsiveness, and increased mortality (Rodriguez and Parra-Lopez, 2024). These immune alterations fuel inflammaging and are associated with cardiovascular, oncological, and neurodegenerative diseases, underscoring CMV as a central determinant of the biology of aging (Weltevrede et al., 2016).

### 7.2 Human herpesvirus 6 and 7 (HHV-6/7)

HHV-6 comprises two strains: HHV-6A and HHV-6B. While HHV-6B causes roseola and is linked to severe neurological conditions and transplant failure (Jaworska et al., 2010), the role of HHV-6A is less understood. However, HHV-6A has been detected at higher levels during relapses in a subset of relapsing-remitting MS patients (Álvarez-Lafuente et al., 2006) and, along with HHV-7, shows increased levels in multiple brain regions of AD patients (Readhead et al., 2018).

At the mechanistic level, HHV-6 reactivation may be facilitated by age-related immune decline and epigenetic drift. Viral genome integration into host telomeres, a hallmark of HHV-6 biology, has been linked to genomic instability, telomere dysfunction, and premature cellular senescence (Arbuckle et al., 2010; Pantry and Medveczky, 2017), potentially contributing to accelerated aging phenotypes. Moreover, chronic low-level HHV-6/7 activity has been implicated in persistent activation of innate immune pathways, including type I interferon signaling, fueling inflammaging. In neurodegeneration, HHV-6A reactivation has been correlated with enhanced amyloid precursor protein processing and neuroinflammatory cascades in AD brain tissue (Readhead et al., 2018). Overall, HHV-6/7 may represent underrecognized viral pathobionts linking aging, immunity, and neurodegeneration, though further research is needed to determine whether they act as causal drivers or bystanders.

### 7.3 Epstein–barr virus (EBV)

EBV infects nearly the entire global population and establishes latency primarily in B lymphocytes, though it can also infect epithelial cells (Smatti et al., 2018). Most infections remain clinically silent due to effective immune control, although some individuals develop infectious mononucleosis or EBV-driven malignancies. Lifelong latency in memory B cells allows viral persistence, with subclinical reactivations fueling chronic inflammation and overt reactivation triggering lymphoproliferative disorders, autoimmune flares, or frailty in older adults (Hatton et al., 2014; Suzich and Cliffe, 2018; Noronha et al., 2021; Gadoth et al., 2024).

In older adults, EBV-driven antigenic pressure contributes to immune exhaustion, with expansion of dysfunctional T and NK cells expressing inhibitory checkpoints (Mangiaterra et al., 2024; Hu et al., 2025). This impairs both antiviral and tumor immune surveillance, consistent with the higher incidence of EBV-related malignancies in aging. Furthermore, EBV has been linked to late-onset autoimmunity, with evidence associating it with rheumatoid arthritis (Miljanovic et al., 2023) and MS in elderly cohorts (Bjornevik et al., 2023), highlighting EBV as a contributor to immune dysregulation in aging, extending its relevance beyond oncogenesis.

### 7.4 Herpes simplex viruses (HSV-1 and HSV-2)

HSV-1 and HSV-2 are widespread, infecting approximately 64% and 13% of the global population, respectively (Birkmann and Saunders, 2025). These neurotropic viruses establish lifelong latency in sensory ganglia (Bello-Morales et al., 2018; Quadiri et al., 2024). HSV-1 is mainly linked to orolabial lesions and encephalitis, while HSV-2 is associated with genital infections (Hou et al., 2017). In older adults, latency becomes increasingly destabilized: impaired type I interferon responses, suboptimal dendritic cell function, and reduced virus-specific CD8^+^ T cell activity contribute to more frequent and severe reactivations (Srivastava et al., 2025).

Epidemiological and mechanistic studies further link HSV-1 reactivation to neurodegeneration, with recurrent viral episodes driving β-amyloid deposition, tau hyperphosphorylation, and neuroinflammation, thereby implicating HSV-1 as a potential pathobiont in AD within the broader framework of the aging virome (Protto et al., 2024; Cairns et al., 2025).

### 7.5 Varicella-zoster virus (VZV)

VZV, the etiological agent of herpes zoster (HZ), establishes lifelong latency in sensory ganglia after primary infection in childhood (Zerboni et al., 2014). Age-related immune decline can trigger reactivation, leading to HZ, which is associated with prolonged morbidity, including post-herpetic neuralgia, sensory deficits, and increased risk of stroke and myocardial infarction (Zhang et al., 2017; Curran et al., 2023; Bakradze et al., 2019). In older adults, T cell–mediated immunity against VZV declines, with expansion of senescent and exhausted VZV-specific CD4^+^ and CD8^+^ memory T cells despite preserved interferon-γ responses (Mangmee et al., 2025). HZ incidence rises with age, reflecting progressive loss of immune surveillance (Muñoz-Quiles et al., 2020). Vaccination with the recombinant zoster vaccine (RZV, Shingrix) provides durable protection, approximately 80% up to 10 years and ∼73% in adults ≥70 years, by sustaining VZV-specific T cell immunity and reducing subclinical viral reactivation (Strezova et al., 2022, 2025; Williams et al., 2025). Beyond preventing HZ, RZV exemplifies a Geroscience-based approach, mitigating chronic antigenic stimulation and inflammaging while enhancing immune resilience in aging populations.

### 7.6 The epigenetics of herpesvirus latency in the aging host

Herpesvirus latency is governed by epigenetic mechanisms that progressively weaken with age. In HSV-1, the viral genome is maintained in heterochromatin (H3K9me3, H3K27me3); however, age-related oxidative stress and the decline of interferon responses compromise this silencing, while neuronal miRNAs regulating latency (*e.g.*, miR-9) also become dysregulated (Messer et al., 2015; Tsai et al., 2022; Deng et al., 2024). In CMV, repression of the major immediate-early promoter by CCCTC-binding factor and bromodomain and extra-terminal domain proteins is relaxed during myeloid differentiation or under inflammatory conditions, facilitating viral reactivation (Groves et al., 2021; Groves et al., 2024). EBV latency programs rely on 3D chromatin topology and viral cofactors such as EBNA-LP, which are perturbed by age-related epigenetic drift, promoting oncogenic gene expression (Caruso et al., 2023; Lieberman and Tempera, 2025). In VZV, latency is maintained by minimal transcription of the VZV latency-associated transcript (VLT)/VLT-ORF63 under tight epigenetic control, which becomes less stable with age, increasing the likelihood of reactivation (Ouwendijk et al., 2020).

### 7.7 Emerging therapeutic and preventive strategies for herpesvirus infections in older adults

Herpesviruses are emerging as key targets of Geroscience-oriented interventions. The RZV remains the most successful example, providing durable protection against HZ and postherpetic neuralgia, while reducing inflammation from VZV reactivation (Strezova et al., 2022; Williams et al., 2025). For CMV, antivirals such as Letermovir and Maribavir have demonstrated reduced toxicity in frail populations (Shigle et al., 2020; Walti et al., 2023). EBV vaccine candidates (*e.g.*, mRNA-1189) aim to prevent infectious mononucleosis and long-term immune complications, while HSV-1 latency-targeted approaches, including CRISPR-based genome editing and epigenetic inhibitors, are under investigation to reduce latent genomes or prevent viral reactivation (Amrani et al., 2024; Aubert et al., 2024).

Taken together, these findings demonstrate that, in older adults, viral pathobionts, including endogenous retroelements and herpesviruses, can profoundly influence aging, by promoting persistent immune activation, inflammaging, and immunosenescence. Understanding the molecular and epigenetic mechanisms that govern their latency and developing strategies to prevent reactivation in the setting of age-related immune decline, represent critical opportunities to foster healthier and more resilient aging trajectories.

## 8 Current challenges in virome profiling: technical and bioinformatic limitations

Despite the growing interest in the role of the virome in health, aging, and longevity, its comprehensive characterization remains a significant challenge. Several technical, analytical, and computational factors limit the accurate identification and interpretation of viral sequences, especially in complex microbial environments such as the gut or bloodstream. In particular, issues related to sequencing protocols and platform sensitivity, as well as the incompleteness and bias of available virome reference databases, hinder our ability to reconstruct viral diversity and dynamics. The following two sections outline the main obstacles in virome research, focusing first on the technical and analytical challenges of sequencing, and then on the current limitations of reference databases.

### 8.1 Technical and analytical pitfalls in virome enrichment and molecular approaches

The NGS technologies have prompted metagenomic studies over the last two decades, greatly increasing our understanding of how microorganisms influence human physiology and disease. Although most of the studies to date focused their attention on the bacterial counterpart, accumulating evidence indicates that the virome also contributes to human health and disease. However, it should be underlined that virome research poses distinct analytical challenges that must be considered during both study design and data analysis. Bacterial communities can be profiled through 16S rRNA amplicon sequencing, a simple and cost-effective approach that has generated large datasets and facilitated extensive characterization of the bacterial microbiome. In contrast, viruses lack a universal phylogenetic marker and require large-scale metagenomic approaches, which increase sequencing time and costs (Garmaeva et al., 2019; Sandybayev et al., 2022). Pre-analytical steps (collection, transport and storage) are critical to avoid contamination, an issue that is especially acute for low-biomass samples such as those used to profile viral communities. Contaminant DNA and cross-contamination can profoundly bias results; best practices and checklists have been proposed to mitigate these risks (Eisenhofer et al., 2019). In addition, DNase treatment, commonly used to remove non-encapsidated DNA, can inadvertently degrade viral genomes that are not fully protected within stable capsids, thereby introducing further bias in virome preparation. Once carefully collected, samples should be processed immediately for DNA extraction or, alternatively, frozen on dry ice and stored at −80 °C. This procedure helps preserve, and effectively “lock”, the microbial communities and their relative abundances at the time of collection; otherwise, ongoing microbial interactions may alter the sample composition, making it no longer representative of the *in vivo* state (Shkoporov et al., 2018).

Nucleic acid extraction represents another critical step. Viral communities can be characterized either through shotgun metagenomic approaches, which rely on the extraction of total DNA from the sample, or by focusing on isolated viral particles. Whole metagenomes are dominated by bacterial sequences, with viral reads often representing only a minor fraction, which explains why enrichment strategies are frequently required to obtain sufficient viral signal for virome sequencing. Approaches targeting isolated viral particles rely on enrichment procedures, such as ultracentrifugation or chemical precipitation, each of which introduces specific methodological biases (Manrique et al., 2016; Ramirez-Martinez et al., 2018; Mirzaei et al., 2021).

Another class of viral contaminants from laboratories environment are known as “kitome” and derive from spin-columns and other extraction kit reagents (Sabatier et al., 2020). These reagents-derived reads can significantly alter taxonomic profiling results. Regardless of these methodological differences, enrichment of viral particles offers the advantage of specifically targeting the viral fraction, thereby reducing the need for deep sequencing (with a positive impact on costs) and simplifying downstream bioinformatic analyses. However, this approach often involves multiple time-consuming steps, and the final yield may be insufficient for subsequent library preparation (Garmaeva et al., 2019). Phages typically represent the main component of virome studies. When total metagenomic DNA is extracted, however, the resulting dataset is overwhelmingly dominated by bacterial sequences, and viral reads often represent only a very small fraction. For this reason, most virome studies rely on enrichment approaches targeting viral particles, rather than on whole-metagenome sequencing, to obtain a sufficient viral signal for meaningful characterization. Nevertheless, most virome studies to date have relied on DNA-based approaches, potentially underestimating the role of RNA viruses. Similarly, single-stranded DNA viruses are often underrepresented due to challenges in their isolation and amplification biases (Roux et al., 2016). Genomic library preparation can be particularly challenging for low-biomass samples, such as those obtained from viral particle enrichments. In these cases, whole-genome amplification may be employed to increase yield, although this approach can introduce methodological biases (Garmaeva et al., 2019; Wang et al., 2023). For RNA and ssDNA viruses, additional steps are often required and should be optimized according to the sample type and the specific objectives of the study. Finally, sequencing depth must be carefully evaluated to ensure sufficient coverage for a comprehensive characterization of the metagenome and for the assembly of the highly diverse and often low-abundance viral fractions. Adequate depth is essential to recover complete or near-complete viral genomes, including its less abundant viral fractions. Emerging long-read sequencing technologies may help overcome current limitations by reducing assembly biases, while direct RNA sequencing approaches—by bypassing cDNA synthesis and its associated biases—hold promise for more accurate investigation of RNA viruses (Smith et al., 2022). The gut virome is extremely diverse, encompassing a wide range of viral morphologies, genome types, and sequence content. This diversity is a major driver of methodological biases, and no single approach can fully capture the virome, highlighting the need for complementary strategies to achieve a more complete characterization. In addition, it is important to acknowledge that enrichment-based approaches themselves introduce biases. Methods that isolate viral particles often select viruses based on physical characteristics such as size, capsid stability, or density, potentially underrepresenting or excluding certain viral taxa (Parras-Molto and Lopez-Bueno, 2018). Moreover, virome sequencing typically captures extracellular viral particles and thus does not account for integrated prophages, which are also poorly characterized in metagenomes and represent a particularly challenging component of the virome to study.

Taken together, these pitfalls highlight why, despite remarkable advances in sequencing technologies, our understanding of the human virome still lags far behind that of the bacterial microbiome. Overcoming these challenges will require not only technical improvements but also careful consideration of how methodological choices at every stage can profoundly influence biological interpretations.

### 8.2 Virome database limitations

While many of the findings discussed in previous sections are derived from metagenomic analyses, current virome databases remain incomplete, biased, and lack standardized protocols for data processing and interpretation. This limits the resolution with which we can explore the diversity, function, and host interactions of viral communities, particularly in aging populations where subtle shifts in virome composition may have significant biological consequences. These issues are exacerbated by the extreme sequence diversity of viruses and by the limited sampling available across ecosystems, which makes it difficult to compare viral sequences across studies and highlights the lack of a centralized, comprehensive viral database.

Recent studies have emphasized the substantial difficulties involved in detecting viral sequences within complex, mixed-community datasets, a task that remains notoriously problematic (Gregory et al., 2020). Major obstacles include the lack of standardized analytical pipelines, inconsistencies in sample processing, the vast underrepresentation of viral genomes in reference databases, the limited availability of culturable host microbes, and pronounced inter-individual variability. These challenges are even more pronounced for phage-specific databases, which remain particularly sparse, poorly curated, and less frequently updated. Together, these factors contribute to a fragmented and often incomplete picture of the human virome and its contribution to health and disease during aging. In addition, many predicted viral ORFs encode hypothetical proteins, making functional inference particularly challenging due to the scarcity of homologs in current reference databases.

A further complication arises from database misclassification. In 2022, Chen et al. (2022) identified about 2 million exogenous sequences annotated as viral sequences in the Reference Sequence database, GenBank, and non-redundant databases. Authors found misclassified entries deriving from host genomic fragments, bacterial DNA and artificially designed vectors. Among laboratory-derived contaminants, they detected sequences containing functional cassettes originating from Simian Virus 40, CMV promoters and retroviral gag (group-specific antigen) and polymerase elements.

To prevent false positives in viromic profiles curated reference resources such as the Eukaryotic Viral Reference Database (EVRD) should be preferred. Alternatively, potential contaminants can be removed using additional controls, such as reference vector databases or tools like UniVec (The UniVec Database) and VecScreen (Schäffer et al., 2018).

Finally, even high-quality reference databases must contend with the lack of sequences from understudied species and with ongoing revisions in viral taxonomy by the International Committee on Taxonomy of Viruses. For example, the ICTV Master Species List release of 2021 updated the *Orthonairoviridae* family with 26 new species and renamed numerous species in pre-existing vOTUs (Relich and Loeffelholz, 2023). Moreover, the 2025 ICTV update introduced major revisions to phage taxonomy, further emphasizing the instability of current viral classification frameworks. In addition, phage–host linkage cannot be directly inferred and requires additional analyses (*e.g.*, CRISPR spacers, co-abundance signals), which may provide limited or no results. To address both limitations, modern bioinformatics pipelines usually consider both reference-based classifiers and reference-free machine-learning approaches. Kraken, a taxonomic classifier for genomic and metagenomic sequences, relies on exact matches of k-mers (subsequences of length k from DNA or RNA sequences) against large nucleotide repositories to rapidly annotate known viruses. However, it cannot detect novel clades absent from its database (Wood and Salzberg, 2014). In contrast, reference-free tools such as VirFinder identify distinguishing k-mer signatures using logistic regression (Ren et al., 2017), while DeepVirFinder leverages convolutional neural networks trained on k-mer encodings to improve accuracy on ∼300-bp fragments (Ren et al., 2020).

Despite increasing efforts to curate and clean up viral reference sets, a large portion of reads in virome studies, ranging from 60% to 90%, remains taxonomically unassigned and it is commonly termed “viral dark matter” (Roux et al., 2015; Santiago-Rodriguez and Hollister, 2022). This gap reflects not only the lack of viral genomes in public databases but also the rapid evolution and ecological heterogeneity of viruses in nature. Together with the poor sampling of viromes across hosts and environments, these factors continue to limit our ability to compare viral communities across studies and age groups.

### 8.3 Challenges in disentangling causality in virome-ageing interactions

A further conceptual limitation in virome research is the difficulty in determining whether virome alterations observed in aging act as causal drivers of biological decline, reflect underlying immune dysfunction, or represent epiphenomena of bacterial dysbiosis. Ageing is accompanied by immunosenescence and chronic inflammation (Gadoth et al., 2024; Noronha et al., 2021; Cianfruglia et al., 2025; Giacconi et al., 2023), both of which may influence viral reactivation patterns and favor shifts in gut and circulating viromes (Jergovic et al., 2019; Mayneris-Perxachs et al., 2022; Yang et al., 2021; Lai et al., 2023). At the same time, age-related changes in the bacteriome, including reductions in *Firmicutes* and increases in Proteobacteria, strongly shape phage community structure (Francini et al., 2025), complicating the interpretation of phage expansions, which may either contribute to dysbiosis or simply mirror bacterial fluctuations (Fujimoto and Daichi, 2022; Yang et al., 2021). Because these processes occur simultaneously, cross-sectional virome studies cannot resolve temporal or causal relationships. Disentangling these competing hypotheses will require longitudinal sampling, multi-omic integration, and models capable of investigating mechanistic phage-host interactions (Shkoporov, 2019). Until such evidence is available, virome changes in ageing should be interpreted with caution, acknowledging both causal and reactive scenarios.

## 9 Conclusion

In recent years, the virome has emerged as a key, even though still underexplored, component of the human microbiota, with profound implications for ageing and longevity. The virome actively shapes host physiology through complex interactions with the bacteriome, the immune system, and brain-gut axis. Emerging evidence indicates that age-associated shifts in the composition and function of both the gut and circulating virome contribute to chronic inflammation, immune remodelling, and neurodegeneration hallmarks unhealthy ageing.

Endogenous and latent viruses, such as HERVs and herpesviruses, exemplify the concept of viral pathobionts: generally tolerated in youth, yet prone to reactivation and immunopathogenicity in later life. These interactions may influence host resilience, cognitive function, and disease risk, underscoring the virome’s potential as both a biomarker and therapeutic target in Geroscience.

The standardisation of analytical pipelines, expansion of viral reference databases, and functional validation of host–virus interactions are urgently required. The extreme diversity of the gut virome, in sequence content, morphologies, and genome types, is a major driver of methodological biases and underscores that no single approach can capture the full virome; complementary strategies are therefore necessary for comprehensive characterization. Longitudinal studies are particularly necessary to establish causality and to capture the dynamic evolution of the virome across the lifespan.

Importantly, the virome also holds promise as a tool for modelling the gut bacteriome, providing innovative strategies to restore microbial balance and may counteract antibiotic resistance. Ultimately, the virome may represent a critical missing piece in the puzzle of longevity, offering both diagnostic and therapeutic opportunities to extend healthspan.
